# BDNF Val66Met polymorphism is associated with altered activity-dependent modulation of short-interval intracortical inhibition in bilateral M1

**DOI:** 10.1371/journal.pone.0197505

**Published:** 2018-06-01

**Authors:** Olivier Morin-Moncet, Alexandre Latulipe-Loiselle, Jean-Marc Therrien-Blanchet, Hugo Theoret

**Affiliations:** 1 Université de Montréal, Montréal, Canada; 2 Hôpital Sainte-Justine Research Center, Montréal, Canada; University of Ottawa, CANADA

## Abstract

The BDNF Val66Met polymorphism is associated with impaired short-term plasticity in the motor cortex, short-term motor learning, and intermanual transfer of a procedural motor skill. Here, we investigated the impact of the Val66Met polymorphism on the modulation of cortical excitability and interhemispheric inhibition through sensorimotor practice of simple dynamic skills with the right and left first dorsal interosseous (FDI) muscles. To that end, we compared motor evoked potentials (MEP) amplitudes and short-interval intracortical inhibition (SICI) in the bilateral representations of the FDI muscle in the primary motor cortex (M1), and interhemispheric inhibition (IHI) from the left to right M1, before and after right and left FDI muscle training in an alternated sequence. Val66Met participants did not differ from their Val66Val counterparts on motor performance at baseline and following motor training, or on measures of MEP amplitude and IHI. However, while the Val66Val group displayed significant SICI reduction in the bilateral M1 in response to motor training, SICI remained unchanged in the Val66Met group. Further, Val66Val group’s SICI decrease in the left M1, which was also observed following unimanual training with the right hand in the Control Right group, was correlated with motor improvement with the left hand. The potential interaction between left and right M1 activity during bimanual training and the implications of altered activity-dependent cortical excitability on short-term motor learning in Val66Met carriers are discussed.

## Introduction

Brain-derived neurotrophic factor (BDNF) is abundant throughout the brain [1, 2]. BDNF is largely synthesized through the activation of glutamatergic neurons and modulates pre- and post-synaptic plasticity by regulating glutamatergic excitatory and gamma-Aminobutyric acid (GABA) inhibitory transmission [1–9]. Thus, it is perhaps not surprising that BDNF is involved in long-term potentiation (LTP) and depression (LTD) [6, 8, 10–16], two mechanisms of cortical plasticity that are associated with learning in the primary cortex (M1) [17–20]. While the most frequent variant of the BDNF gene is Val66Val, recent studies have shown that a single nucleotide polymorphism of the BDNF gene at codon 66 (Val66Met), found in approximately one third of the American population [21], relates to reduced BDNF activity-dependent expression, altered glutamatergic and GABA-ergic synaptic transmission, and altered white matter integrity in the corpus callosum [22–25]. Incidentally, Val66Met carriers exhibit impaired M1 cortical excitability, short-term motor learning, retention and intermanual transfer of a motor skill [26–29].

Transcranial magnetic stimulation (TMS), a non-invasive brain stimulation technique, is widely used to assess activity-dependent changes in intracortical and corticospinal excitability that occur through motor practice [30–35]. Motor Evoked Potentials (MEP) reflect the global changes in cortical excitability via the direct and indirect stimulation of corticospinal fibers as well as spinal cells [20, 36–38]. In addition, GABA-mediated intracortical inhibition mechanisms can be assessed with paired pulse stimulation techniques using short latency and sub-threshold pre-conditioning in M1, in a process known as short-interval intracortical inhibition (SICI) [32, 39–43]. Both processes are particularly relevant to manual motor learning and transfer as studies have repeatedly shown, although not systematically [44], increases in MEP amplitudes and decreases in SICI in the M1 contralateral to the trained hand following motor practice, and reduced SICI in the transfer hemisphere [39, 45–47]. Unilateral hand movements with the dominant or the non-dominant hand may also activate bilateral M1 and alter cortical excitability (MEP; SICI) in the homotopic muscle representations in the ipsilateral M1, more so when the non-dominant hand is activated [48–56]. These observations highlight the interhemispheric interactions that occur during motor practice, where the hemisphere contralateral to the trained hand inhibits superfluous activity originating from the opposite hemisphere in a process known as interhemispheric inhibition (IHI) [52, 57–61]. Recent studies have suggested IHI involvement in the intermanual transfer of procedural and sensorimotor learning [45, 47, 62]. Despite advancements on the possible influence of the Val66Met polymorphism on M1 corticospinal excitability, motor learning and intermanual transfer of procedural motor skills, it remains unclear whether the Val66Met polymorphism interferes with activity-dependent modulation of neurophysiological mechanisms that underlie their expression, namely SICI and IHI.

The first objective of the present study was to assess motor learning of low-force sensorimotor skills with the right hand, and their intermanual transfer from the right hand to the left hand, in Val66Val and Val66Met participants. Rapid Tapping (RT), Precision Grip Strength (PGS), and Grooved Pegboard (GPB) tasks were used, as intermanual transfer of these motor skills has been shown in separate experiments [63–67], and since differences in corticospinal excitability in the contralateral M1 has been evidenced between both groups following motor training on similar tasks [26, 28]. Given the absence of reported differences in motor learning on tasks involving sensorimotor adaptations between Val66Val and Val66Met individuals [26–28], it was hypothesized that motor learning with the right, dominant hand would not differ between groups. However, in light of recent data showing reduced intermanual transfer of a procedural motor skill after a single session of motor training in individuals with the Val66Met genotype [29], it was hypothesized that participants with the Val66Met polymorphism would show impaired intermanual transfer of sensorimotor skills from the right, dominant to the left, non-dominant hand.

The second objective of the present study was to compare neurophysiological mechanisms that underlie motor learning and the intermanual transfer of sensorimotor skills, namely corticospinal excitability and SICI in bilateral M1, and IHI from the left to right M1. It was hypothesized that participants with the Val66Met polymorphism would display reduced modulation of MEP and SICI in the bilateral M1, and IHI from the left to right M1, compared to Val66Val participants.

## Materials and methods

### Participants

#### Experiment 1

Fifty-six right-handed participants aged 18 to 35 years old were recruited for genotyping. Out of the fifty-six participants, thirteen presented the Val66Met polymorphism, three males and ten females. The three males and seven randomly selected females were assigned to the Val66Met group (21.90±2.28 years, 7 women). Participants in the Val66Val group were matched on gender to control for known interactions between the Val66Met polymorphism and gender on motor control [68]. Thus, three males and seven females were randomly selected and assigned to the Val66Val group (21.90±1.79 years, 7 women). In addition, ten right-handed participants were separately recruited and assigned to the Control-Left group (CL; 23.10±3.75 years, 7 women). The CL group was used to isolate the behavioral effects of motor practice with the left hand to determine the presence of intermanual transfer in the experimental groups who performed the motor tasks with both hands in an alternated sequence.

#### Experiment 2 (control)

Ten right-handed participants were recruited in a separate experiment and assigned to the Control-Right group (CR; 24.60±3.77 years, 7 women). This control experiment served two functions: *i)* show that the tasks used in Experiment 1 and executed with the right hand elicited motor learning; and *ii*) show that the tasks used in Experiment 1 and executed with the right hand induced modulation of MEP amplitudes and SICI in the left M1, and IHI from the left to right M1, in the expected directions.

All participants declared being healthy and reported no history of psychiatric or neurological condition, and none presented with contraindications to the safe use of TMS [69]. They provided their written informed consent to undergo experimental procedures approved by the Comité d’Éthique de la Recherche des Sciences de la Santé (CÉRSS) and compliant with the 1964 Declaration of Helsinki.

### Genotyping

The BDNF genotyping method was identical to the procedure described in Morin-Moncet et al. (2014). Briefly, DNA was extracted from the saliva with Oragene OG-250s kits (DNA Genotek, Ottawa, ON, Canada). PCR following an established pyrosequencing protocol [70] was performed to determine the profiling of the BDNF rs6265 (Val66Met) polymorphism.

### Behavioral tasks

**Fig 1:**
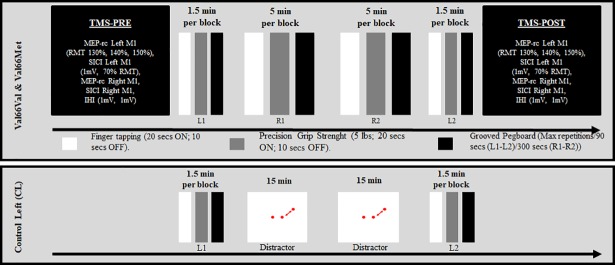
Experiment 1. The experimental design used in Experiment 1 is displayed for the Val66Val, the Val66Met, and the Control-Left (CL) groups.

Participants were seated comfortably in an upright position facing a computer screen at approximately three feet with the elbows flexed at a 90° angle and the wrists resting on a table. The subjects were instructed to perform a series of three motor tasks in a fixed order: RT, PGS, and GPB (see Fig 1). Motor performance with the left hand was measured in two conditions, L1 and L2. L1 was used as a pre-training, baseline measurement of motor performance. L2 served as a measurement of performance following right hand training (R1, R2). L1 and L2 were identical and consisted of three blocks of the RT and the PGS tasks, lasting 30 seconds each, followed by 90 seconds of continuous GPB, for a total of approximately 5 minutes per condition.

Motor training with the right hand comprised two identical conditions, R1 and R2, to assess motor learning. Both conditions consisted of ten blocks of the RT and PGS tasks, followed by five minutes of continuous GPB, for a combined R1 and R2 time of 30 minutes. For the three tasks, a green and red circle appeared in the middle of a computer screen, accompanied by a sound signal, to indicate the Go and Stop signals, respectively. The signal presentation and the duration of the blocks were monitored by Presentation software (Neurobehavioral Systems ®, California, USA).

**Fig 2:**
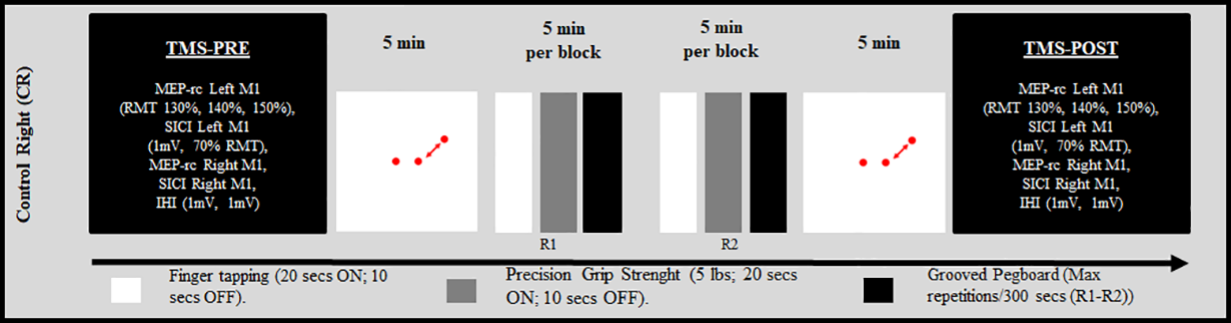
Experiment 2. The experimental design used in experiment 2 is displayed for the Control-Right (CR) group.

To control for the learning effects resulting from practice with the left hand during L1 and L2, the CL group performed the L1 and the L2 conditions only, separated by a 30-minute period during which participants performed a computerized distractor task. In Experiment 2, the CR group executed R1 and R2 blocks with 5 minutes of the computerized distractor task before and after right-hand training (see Fig 2). The distractor task was run with Psyscope X software running on a 17-inch I-Mac computer (Apple, Cupertino, USA). It was designed to ensure sustained focus and limited motor activity. The task consisted in the presentation of two red dots symmetrically located across the center of the screen. The left dot was static while the right dot moved in a semi-circular, counter-clockwise 90° rotation, and back to its original position, at a frequency of 0.5 hertz. Participants were asked to silently count the number of rotations, which varied between 25 and 35 in each block.

Rapid Tapping (RT). Participants were instructed to alternate key presses of the 1 (index finger) and 3 (middle finger) keys on a numeric keypad as fast as possible. Reaction times, representing delays in milliseconds between alternated key presses with the index and the middle finger, were recorded using Presentation software (Neurobehavioral Systems ®, California, USA) and stored off-line for analysis. A single block was defined as 20 seconds of finger tapping followed by 10 seconds of rest for a total of 30 seconds. Reaction times were averaged for the total number of blocks for each condition (L1, R1, R2, L2).

Precision Grip Strength (PGS). Participants were instructed to apply and maintain, as precisely as possible, a pressure of 5lbs using their index and middle finger on a pinch gauge dynamometer (JTech Commander ®, Utah, United States). A single block consisted of 20 seconds of pinch pressure followed by 10 seconds of rest, for a total of 30 seconds. The precision score was computed as the average of the absolute difference between the pressure score and the target score (5lbs) at each second (1 Hertz) for the total number of blocks on each condition (L1, R1, R2, L2).

Grooved Pegboard (GPB). Two original Grooved Pegboard Tests (Lafayette Instruments ®, Indiana, USA) were used for this task. Participants were instructed to fill up the rows from left to right and top to bottom, one peg at a time, as fast as possible, using their index finger and thumb. They were instructed to ignore dropped pegs and to proceed with the second GPB upon completion of the first. A block consisted of 90 seconds of continuous execution in the L1 and L2 conditions, and in five minutes of continuous execution on the R1 and R2 conditions. A dexterity score was computed as the average time in seconds taken to complete each row, per condition (L1, R1, R2, L2).

### Transcranial magnetic stimulation

TMS pulses were delivered using two 8-cm figure-of-eight coil connected to a Magstim BiStim (Magstim company, Whitland, Wales, UK). The stimulation coils were held posterolaterally at a 45° angle from the midline and applied flat on the scalp. For the MEP recruitment curves (MEP-rc) and SICI, a brainsight neuronavigating system (Rogue Research, Montréal, Canada) was used to ensure stable positioning of the coil throughout the experiment. For IHI, the optimal stimulation site of the left FDI muscle over the right M1 was marked on the participants’ scalp. MEPs were recorded using bipolar surface electrodes positioned over the left and right FDI muscles, and the ground electrode positioned on the right inner forearm. The electromyographic signal was amplified using a Powerlab 4 ⁄ 30 system (ADInstruments, Colorado Springs, USA; gain factor: 1000), filtered with a band-pass 20–1000 Hz, and digitized at a sampling rate of 4 kHz. MEPs were recorded using Scope v4.0 software (ADInstruments, Colorado Springs, USA) and stored offline for analysis. The optimal stimulation site for the bilateral first dorsal interosseous (FDI) muscle representation in M1 was defined as the area on the scalp eliciting MEPs of maximal amplitude in the left and right FDI muscles. Complete relaxation of the FDI muscle was controlled by monitoring the absence of EMG signal up to 100 ms before the TMS pulse. The number of MEPs excluded was marginal (i.e. less than 1%). TMS pulses were delivered with 6–7 seconds inter-stimulus intervals to avoid anticipation. Resting motor threshold (RMT) was determined prior to both TMS sessions, before (session 1: Pre) and after (session 2: post) motor training. RMT was defined as the minimum intensity level of TMS required to elicit MEPs of 50 μV in at least 5 trials out of 10 [71]. Further, the TMS intensity required to elicit MEP amplitudes of ≈ 1 mV on average for ten stimulations at rest was determined before the SICI measurements in the left and right M1. MEP-rc and SICI were measured in sequence, pre- and post- motor training beginning with the left M1. IHI from the left to right M1 was measured last, Pre- and Post- motor practice (see Fig 1).

MEP-rc. Corticospinal excitability was measured first, pre- and immediately post- motor training. The MEP-rc consisted of ten TMS pulses delivered at 130, 140, and 150 percent of the participants’ RMT in a semi-randomized order, with an interstimulus interval of 6–7 seconds, for a total of 30 MEPs. These intensities were selected based on previous work on intermanual transfer of motor skill showing significant increases in MEP amplitudes at these intensity levels compared to intensities ≤ 120% RMT [45, 47]. Peak-to-Peak MEP amplitudes were measured and averaged for each intensity level, pre- and post- motor training.

SICI and IHI. For SICI, paired pulse stimulation was delivered with a conditioning stimulus (CS) intensity of 70% of RMT and a test stimulus (TS) intensity at the level required to elicit MEP amplitudes of ≈ 1 mV at rest, with a 3-millisecond interstimulus interval (ISI). While intracortical facilitatory circuits may interfere with SICI at ISIs greater than 2 milliseconds [72], this ISI was selected in accordance with previous experiments [41, 45, 73] to differentiate between the two phases of inhibition observed at ISIs of 1 millisecond and 2.5 milliseconds, with the latter ISI likely reflecting intracortical GABA-ergic circuitry [32, 74, 75]. IHI from the left to right M1 was assessed with paired CS and TS stimulus intensities set at the level required to elicit MEPs of ≈ 1 mV at rest, over the left and right M1, with an ISI of 10 milliseconds. Ten TS were delivered first, followed by ten CS-TS paired-pulse stimulations. Peak-to-peak MEP amplitudes were averaged separately for the ten TS MEPs and the ten conditioned MEPs. SICI and IHI were defined as the percentage of the averaged conditioned MEP amplitudes relative to the averaged TS MEP amplitudes (i.e. M _conditioned MEP_ /M_TS MEP_ x100), pre- and post- motor training.

### Data analysis

#### Experiment 1

Motor learning with the right hand in the Val66Val and the Val66Met groups was assessed with three mixed ANOVAs, one for each task (RT; PGS; GPB), with *condition* (R1; R2) as the within-subjects factor and *group* (Val66Val; Val66Met) as the between-subjects factor.

The presence of intermanual transfer from the right dominant hand to the left non-dominant hand was individually measured for each of the three tasks (RT; PGS; GPB) by comparing the performance in L1 and L2 between the Val66Val, Val66Met, and the CL groups. To that end, three mixed ANOVAs were computed, with *condition* (L1; L2) as the within-subjects factor and *group* (Val66Val; Val66Met; CL) as the between-subjects factor.

RMT and 1 mV stimulus intensities, expressed as a percentage of the device’s maximal stimulation output, were compared pre- and post- motor practice using two mixed ANOVAs with *hemisphere* (Left M1; Right M1) and *time* (Pre; Post) as the within-subjects factors and *group* (Val66Val; Val66Met) as the between-subjects factor.

MEP-rc was compared pre- and post- motor training between the Val66Val and the Val66Met using mixed ANOVAs with *hemisphere* (Left M1; Right M1), *time* (Pre; Post), and *intensity* (MEP-rc 130; MEP-rc 140; MEP-rc 150) as the within-subjects factors and *group* (Val66Val; Val66Met) as the between-subjects factor. A mixed ANOVA was used to compare SICI with *hemisphere* (Left M1; Right M1) and *time* (Pre; Post) as the within-subjects factors and *group* (Val66Val; Val66Met) as the between-subjects factor. IHI from the left to right M1 was assessed between the Val66Val and Val66Met participants before and after motor practice using a mixed ANOVA with *time* (Pre; Post) as the within-subjects factor and *group* (Val66Val; Val66Met) as the between-subjects factor. In addition, a mixed ANOVA with *hemisphere* (Left M1; Right M1) and *time* (Pre; Post) as within-subjects factors and *group* (Val66Val; Val66Met) as between-subjects factor was computed to compare test MEP amplitudes in SICI. A similar procedure was applied for IHI from the left to right M1, without the *hemisphere* factor.

Normality of the standardized residuals distributions of the TMS and behavioral variables used to compare Val66Val and Val66Met participants was assessed with Kolmogorov-Smirnov and Shapiro-Wilks tests. Non-normal variables were transformed with log10 computations and were reanalyzed with the same procedure as described above.

Finally, intraclass correlation coefficients (ICC) were measured for TMS variables (e.g. RMT: MEP-rc; SICI; IHI) in Val66Val and Val66Met groups separately to insure both samples representativeness based on intersession reliability scores in other experiments [76–80]. Intersession ICCs were obtained with a two-way mixed-effects model. ICCs were interpreted as follows: Excellent: ICC > 0.75; Good: 0.75 ≥ ICC ≥ 0.59; Fair: 0.58 ≥ ICC ≥ 40; Poor: ICC > 40 [76, 81].

#### Experiment 2

The presence of motor learning in the CR group was assessed by comparing performance on the RT, PGS, and GPB tasks on R1 and R2 conditions with separate paired-samples t-tests. RMT and 1 mV stimulus intensities were compared, before and after motor training in the left and right M1, with a mixed ANOVA with *hemisphere* (Left M1; Right M1) and *time* (Pre; Post) as the within-subjects factors. MEP-rc and SICI were compared Pre- and Post- motor training using a mixed ANOVA with *hemisphere* (Left M1; Right M1), *time* (Pre; Post), and *intensity* (MEP-rc 130; MEP-rc 140; MEP-rc 150) as the within-subjects factors for the MEP-rc, and *hemisphere* (Left M1; Right M1) and *time* (Pre; Post) for SICI. IHI from the left to right M1 was compared before and after practice with a paired-samples t-test. Test MEP amplitudes were compared for SICI using a mixed ANOVA with *hemisphere* (Left M1; Right M1) and *time* (Pre; Post) as within-subjects factors, and with a paired-samples T-test for IHI. Post-hoc analyses were conducted, as necessary, using contrasts with Bonferroni correction on the repeated measures models and with Tukey HSD for between-subjects comparisons. Bilateral Spearman correlations between measures of neurophysiological activity and motor performance were performed as necessary. α = 0.05.

## Results

### Experiment 1

#### Behavioral

**Fig 3:**
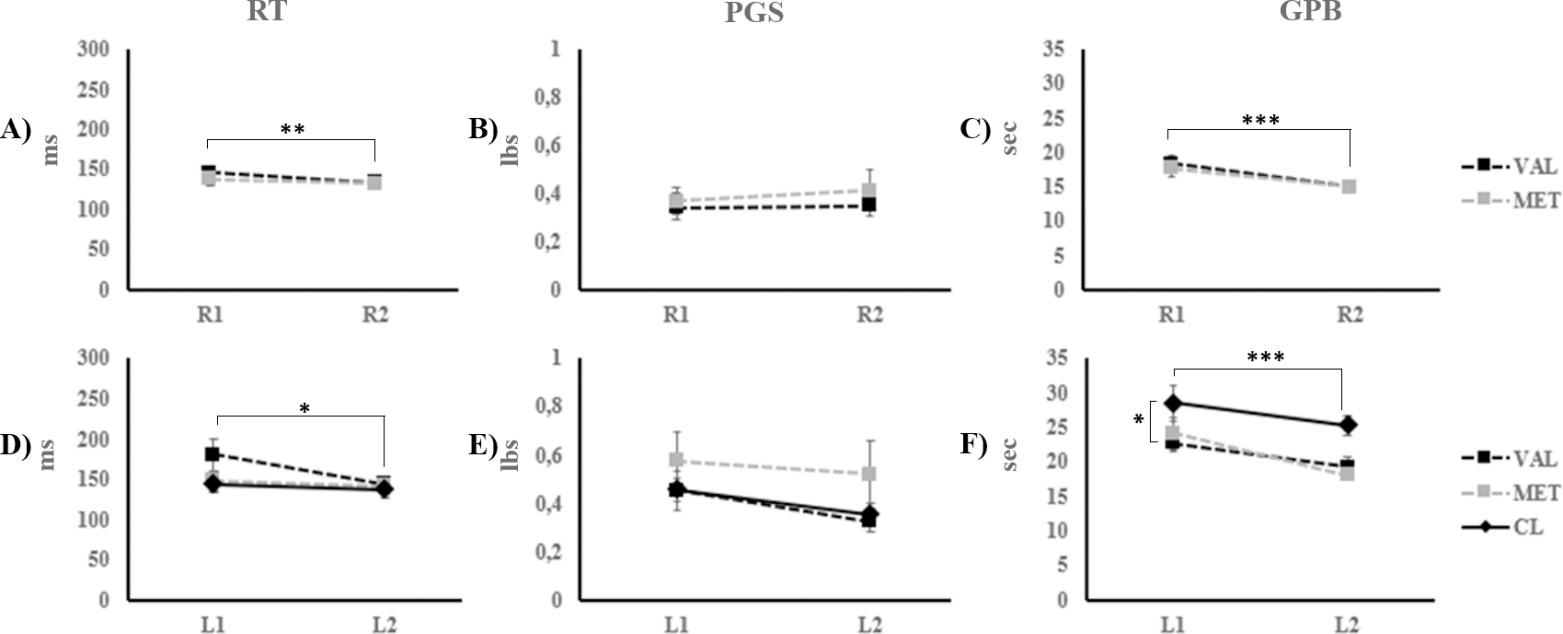
Motor performances on the rapid tapping (RT), pinch grip strength (PGS), and the grooved pegboard (GPB). The mean scores on the RT, the PGS, and the GPB are presented for the Val66Val and the Val66Met participants in Experiment 1 on the R1 and R2 conditions with the right hand (A, B, and C, respectively), and the L1 and L2 conditions with the left hand (D, E, and F, respectively). The error bars represent standard errors of the mean. Full lines used for comparisons represent main effects. * p < 0.05; ** p < 0.01; *** p < 0.001.

Learning with the right hand (see Fig 3A, 3B and 3C). Mixed ANOVAs revealed a significant effect of *condition* on the RT (F_(1, 18)_ = 10.244; p = 0.005) and GPB tasks (F_(1, 18)_ = 32.767; p < 0.001), with no significant effects of *group* (F_RT (1,9)_ = 0.282; p = 0.602; F_GPB (1, 9)_ = 0.598; p = 0.45) or *condition* x *group* interaction (F_RT (1,9)_ = 1.654; p = 0.215; F_GPB (1, 9)_ = 1.629; p = 0.219). For the PGS task, the effects of *condition* (F_(1, 18)_ = 0.448; p = 0.512), *group* (F_(1, 18)_ = 0.11; p = 0.744), and the *condition* x *group* interaction (F_(1, 18)_ = 0.567; p = 0.461) were not significant.

Transfer to left hand (see Fig 3D, 3E, and 3F). For the left-hand conditions, a participant in the Val66Met group was excluded from RT measurements due to technical difficulties during data acquisition. As a result, for the RT task, the CL, Val66Val and the Val66Met groups comprised 10, 10, and 9 participants, respectively. For the RT task, a mixed ANOVA revealed a significant effect of *condition* (F_(1, 26)_ = 7.212; p = 0.012) with no *group* (F_(2, 26)_ = 1.237; p = 0.307), or *condition* x *group* interaction (F_(2, 26)_ = 2.499; p = 0.102) effects. For the GPB task, there was a significant effect of *condition* (F = 16.082; p < 0.001) and *group* (F = 4.399; p = 0.022) while the *condition* x *group* interaction was not significant (F_(2, 27)_ = 0.71; p = 0.499). Post-hoc comparisons revealed slower responses in CL participants compared to Val66Met participants (p = 0.031), with no other difference between groups (all p > 0.05). For the PGS task, the effects of *condition* (F_(1, 27)_ = 3.516; p = 0.072), *group* (F_(2, 27)_ = 1.15; p = 0.332), and the *condition* x *group* interaction (F_(2, 27)_ = 0.219; p = 0.805) were not significant.

In sum, all three groups exhibited improved performance with the left hand on L2 compared to L1 on the RT and the GPB tasks. Moreover, improvements on L2 in the Val66Val and Val66Met groups did not differ from that of the CL group, suggesting an absence of significant skill transfer from the right to the left hand.

#### TMS

RMT and 1 mV intensity. A mixed ANOVA was used to compare RMT intensities before and after motor practice between the Val66Val and Val66Met and revealed a significant effect of *hemisphere* (F_(1, 18)_ = 5.934; p = 0.025), while the effects of *time* (F_(1, 18)_ = 0.488; p = 0.494) and *group* (F_(1, 18)_ = 0.018; p = 0.895) were not significant. Stimulus intensities used to elicit MEPs at the RMT did not differ before and after motor practice, without distinction between genetic groups, and were generally lower in the left M1 compared to the right M1. For 1mV, stimulus intensities did not differ before and after motor practice (F_(1, 18)_ = 2.592; p = 0.125), between the left and right M1 (F_(1, 18)_ = 2.029; p = 0.171), or between groups (F_(1, 18)_ = 0.031; p = 0.862). None of the interactions for the RMT and 1mV measurements were significant (0.04 ≤ F ≤ 1.893; 0.186 ≤ p ≤ 0.844).

**Fig 4:**
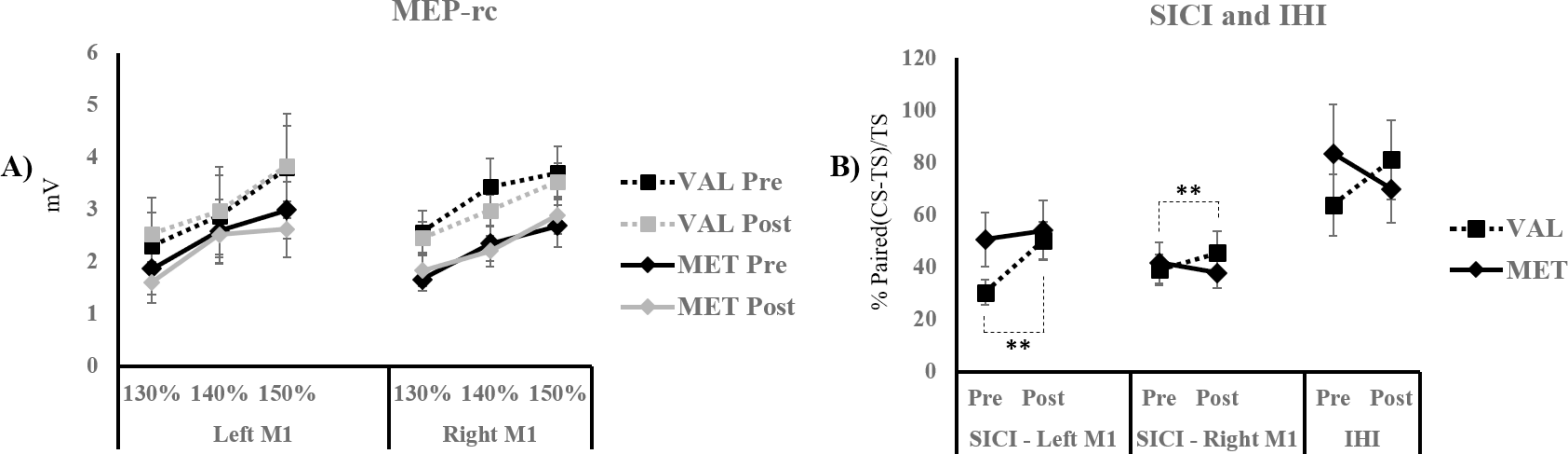
MEP-rc, SICI, and IHI measures. MEP-rc (A), SICI (B), and IHI (B) measures Pre- and Post- motor training are shown for the Val66Val Vs. the Vall6Met groups in the Experiment 1. The error bars represent standard errors of the mean. Dashed lines used for comparisons show simple effects. ** p < 0.01.

MEP-rc (see Fig 4A). A mixed ANOVA showed a significant effect of *intensity* (F_(2, 36)_ = 93.34; p < 0.001). The effects of *time* (F_(1, 18)_ = 0.3; p = 0.591), *hemisphere* (F_(1, 18)_ = 0.001; p = 0.971), *group* (F_(1, 18)_ = 1.584; p = 0.224) or any of the interactions were not significant (0.004 ≤ F ≤ 2.961; 0.074 ≤ p ≤ 0.948).

SICI (see Fig 4B). A mixed ANOVA revealed a non-significant effect of *hemisphere* (F_(1, 18)_ = 1.377; p = 0.256), non-significant interactions involving the *hemisphere* factor (0.12 ≤ F ≤ 2.632; 0.122 ≤ p ≤ 0.733), and a significant *time* x *group* interaction (F_(1, 18)_ = 5.073; p = 0.037; η^2^p = 0.22). Post-hoc contrasts performed on the estimated marginal means for the combined hemispheres with Bonferroni corrections indicated a significant effect of *time* for the Val66Val group (M_Pre_ = 34.68 ± 7.23; M_Post_ = 47.85 ± 6.51; p = 0.006) but not for the Val66Met group (M_Pre_ = 46.11 ± 7.23; M_Post_ = 45.9 ± 6.51; p = 0.959). Between-subjects comparisons of SICI on time 1 and time 2 showed no significant difference in SICI between the Val66Val and the Val66Met Pre (p = 0.278) and Post (p = 0.834) motor practice. Further, test MEP amplitudes in the Val66Val (M_LH-Pre_ = 1.06 ± 0.13 mV; M_LH-Post_ = 1.11 ± 0.34 mV; M_RH-Pre_ = 1.19 ± 0.19 mV; M_RH-Post_ = 1.26 ± 0.25 mV) and the Val66Met (M_LH-Pre_ = 1.05 ± 0.1 mV; M_LH-Post_ = 1.01 ± 0.21 mV; M_RH-Pre_ = 1.19 ± 0.19 mV; M_RH-Post_ = 1.11 ± 0.2 mV) groups did not differ before or after motor practice (F_(1, 18)_ = 0.003; p = 0.960), but they were greater overall in the right M1 compared to the left M1 (F_(1, 18)_ = 11.139; p = 0.004). However, test MEP amplitudes did not differ between groups (F_(1, 18)_ = 1.028; p = 0.324), and none of the interactions were significant (p ≥ 0.275). Therefore, possible effects of TS MEP intensity on conditioned MEPs in bilateral M1 were equivalent between groups.

IHI (see Fig 4B). A mixed ANOVA revealed non-significant effects of *time* (F_(1, 18)_ = 0.017; p = 0.899), *group* (F_(1, 18)_ = 0.073; p = 0.791), or *time* x *group* interaction (F_(1, 18)_ = 1.108; p = 0.307). Test MEP amplitudes also did not differ before or after motor practice (F_(1, 18)_ = 1.028; p = 0.324), nor between groups (Val66Val: M_Pre_ = 1.05 ± 0.22 mV; M_Post_ = 1.2 ± 0.47 mV; Val66Met: M_Pre_ = 1.12 ± 0.3 mV; M_Post_ = 1.11 ± 0.33 mV; F_(1, 18)_ = 0.002; p = 0.918), and the time*group interaction was non-significant (F_(1, 18)_ = 1.337; p = 0.263).

#### Normality tests

Normality analyses of the standardized residual distributions using Kolmogorov-Smirnov and Shapiro-Wilks tests revealed significant deviations from normal distribution on several variables. Specifically, TMS data comparisons between the Val66Val and the Val66Met groups were comprised of non-normal distributions in the MEP-rc residuals for the left M1 on the pre- and post- conditions (0.001 ≤ p ≤ 0.002), in the IHI residuals on the pre- condition (p = 0.021), in the RMT residuals on the right M1’s post- condition (p = 0.031), and in the 1mV residuals in the left (post- condition; p = 0.032) and right M1 (pre- condition; p = 0.033). Likewise, motor performance comparisons between the Val66Val the Val66Met groups yielded significant standardized residual distribution deviations from normality on condition 2 of the PGS task with the right hand (p = 0.03), on condition 1 of the RT task with the left hand (p = 0.027), and on conditions 1 (p = 0.003) and 2 (p = 0.04) of the PGS task with the left hand. As such, log 10 transformations were computed for all variables and the original analyses were performed with the transformed variables. As a result, there were no changes in the significance of the main effects or interactions reported in the original analyses. Further, non-normal standardized residual distributions of the log 10 variables remained, namely in the MEP-rc residuals in the left (p = 0.037) and right M1 (0.014 ≤ p ≤ 0.033), in the IHI residuals on the post- condition (p = 0.032), and in the 1mV residuals in the left (0.018 ≤ p ≤ 0.046) and right M1 (pre- condition; p = 0.028).

#### Intersession reliability

ICCs were excellent for RMT and MEP-rc measures in the left and right M1 at each intensity level in Val66Val (0.863 ≤ r ≤ 0.978; p ≤ 0.002) and Val66Met groups (0.87 ≤ r ≤ 0.963; p ≤ 0.003). For SICI, ICCs were poor for the left M1 (r = 0.27; p = 0.252) and good (r = 0.677; p = 0.056) for the right M1 in the Val66Val group, and excellent (r = 0.892; p = 0.002) and good (r = 0.673; p = 0.063), respectively, in the Val66Met group. In addition, ICCs for IHI from the left to right M1 were fair and invalid, respectively, for the Val66Val (r = 0.484; p = 0.167) and Val66Met groups (r = -0.192; p = 0.593).

### Experiment 2

#### Learning

**Fig 5:**
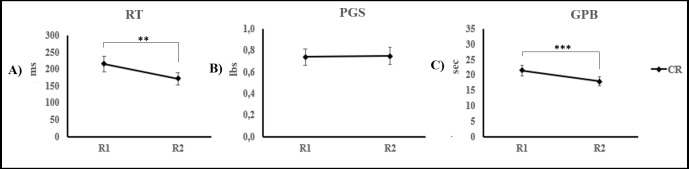
Motor performances on the rapid tapping (RT), pinch grip strength (PGS), and the grooved pegboard (GPB). The mean scores on the RT (A), the PGS (B), and the GPB (C) are presented for the CR participants in Experiment 2 on the R1 and R2 conditions with the right hand. The error bars represent standard errors of the mean. Full lines used for comparisons represent main effects. ** p < 0.01; *** p < 0.001.

In the CR group (see Fig 5A, 5B and 5C), paired-samples t-tests revealed significant speed improvements between R1 and R2 on the RT (M_R1_ = 216 ± 74.74; M_R2_ = 172 ± 60.6; t_(9)_ = 3.758; p = 0.004) and the GPB tasks (M_R1_ = 21 ± 5.6; M_R2_ = 17 ± 4.63; t_(9)_ = 4.5; p = 0.001) but not for the PGS task (M_R1_ = 0.73 ± 0.24; M_R2_ = 0.74 ± 0.26; t_(9)_ = -0.09; p = 0.93).

#### TMS

RMT and 1 mV intensity. Two repeated-measures ANOVAs were used to compare RMT and 1mV intensities before and after motor practice in the CR group’s left and right M1 revealed non-significant effects of *hemisphere* (F_RMT (1,9)_ = 0.596; p = 0.46; F_1mV (1, 9)_ = 0.319; p = 0.586) and *time* (F_RMT (1,9)_ = 0.396; p = 0.545; F_1mV (1, 9)_ = 0.142; p = 0.715). In addition, the *hemisphere* x *time* interaction was not significant (F_RMT (1,9)_ = 1.227; p = 0.297; F_1mV (1, 9)_ = 0.168; p = 0.691).

**Fig 6:**
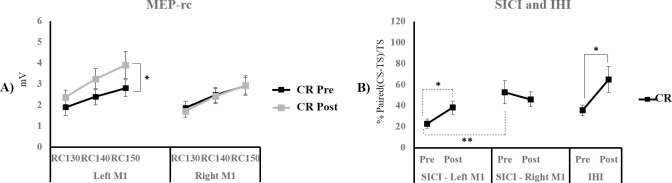
MEP-rc, SICI and IHI measures. MEP-rc (A), SICI (B), and IHI (B) measures Pre- and Post- motor training are shown for the CR group in the Experiment 2. The error bars represent standard errors of the mean. Full lines used for comparisons represent main effects and dashed lines show simple effects. * p < 0.05; ** p < 0.01.

MEP-rc (see Fig 6A). A repeated-measures ANOVA revealed a significant effect of *intensity* (F_(2, 18)_ = 46.106; p < 0.001) and of the *hemisphere* x *time* interaction (F_(1, 9)_ = 9.361; p = 0.014). Post-hoc contrasts conducted on the estimated marginal means of the combined intensity levels using Bonferroni corrections revealed a non-significant effect of *hemisphere* before (M_LeftM1_ = 2.36 ± 0.39 mV; M_rightM1_ = 2.42 ± 0.35 mV; p = 0.918) and after motor practice (M_LeftM1_ = 3.16 ± 0.49 mV; M_rightM1_ = 2.35 ± 0.36 mV; p = 0.155), which suggests that the MEP-rc amplitudes did not differ between the left and right M1. However, the within-subjects comparisons showed a significant increase in MEP-rc amplitudes in the left M1 following motor practice with the right hand (p = 0.025) while MEP-rc amplitudes in the right M1 did not differ before and after motor practice (p = 0.541).

SICI (see Fig 6B). A repeated measures ANOVA revealed a significant *time* x *hemisphere* interaction (F_(1, 9)_ = 5.584; p = 0.042). Post-hoc analysis showed a significant effect of *time* in the left M1 (M_Pre_ = 23.05 ± 4.4; M_Post_ = 52.74 ± 11.13; p = 0.015) but not in the right M1 (M_Pre_ = 38.23 ±6.35; M_Post_ = 46.13 ± 7.1; p = 0.351). In addition, within-subjects comparisons showed a significant effect of *hemisphere* pre- motor practice (p = 0.009), whereas SICI did not differ between hemispheres post motor practice (p = 0.367). Test MEP amplitudes did not differ *pre-* or *post-* motor practice (M_LH-Pre_ = 1.09 ± 0.15 mV; M_LH-Post_ = 0.97 ± 0.19 mV; M_RH-Pre_ = 1.02 ± 0.21 mV; M_RH-Post_ = 1.05 ± 0.32 mV; F_(1, 9)_ = 0.631; p = 0.447), between hemispheres (F_(1, 9)_ = 0.007; p = 0.934), and the time*hemisphere interaction was not significant (F_(1, 9)_ = 1.541; p = 0.246). These results suggest increased SICI before motor practice with the right hand in the left M1 compared to the right M1.

IHI (see Fig 6B). A paired-samples T-test was used to compare IHI from the left to right M1 and revealed a significant decrease following motor practice with the right hand (t_(9)_ = -2.464; p = 0.036). Test MEP amplitudes did not differ before and after motor practice (M_Pre_ = 1.02 ± 0.15 mV; M_Post_ = 0.98 ± 0.26 mV; t_(9)_ = 0.542; p = 0.601).

### Correlation analyses

**Fig 7:**
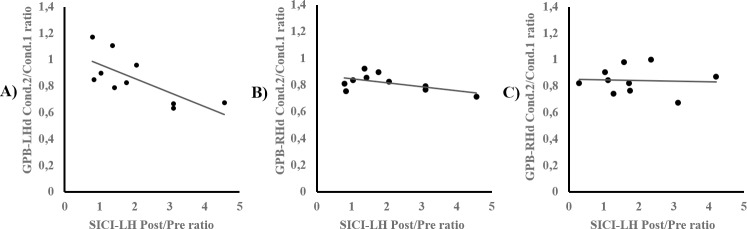
Correlation analyses between SICI modulation in the left M1 and GPB performance. Correlations between Post- to Pre- ratio of SICI in the left M1 (abscissa) and condition 2 to condition 1 ratio of GPB performance (ordinate) are displayed for the Val66Val participants’ left (A) and right (B) hands, and for the CR group’s performance with the right hand (C). Larger SICI ratios indicate a greater decrease in SICI following motor training, and smaller GPB ratios suggest greater improvement from condition 1 to condition 2 on the motor task.

See Fig 7A, 7B and 7C. Relationships between SICI variations pre- and post-motor training, and motor performance improvements from condition 1 to condition 2 on the RT and GPB tasks, were assessed using bilateral Spearman tests in the Val66Val and the CR groups. To that end, Post- to Pre- ratios of SICI were computed for the Val66Val and the CR group’s left and right M1. Likewise, condition 2 to condition 1 ratios of Val66Val and CR participants’ motor performance were performed on the RT and GPB tasks with the right and left hands. In the Val66Val group, Spearman tests revealed non-significant correlations between right M1 SICI ratios and motor improvement ratios on the RT and GPB tasks with the left and right hands (-0.358 ≤ *r*_s_ ≤ 0.588; 0.074 ≤ p ≤ 0.855). SICI ratios in the left M1 were significantly correlated to motor improvement on the GPB task with the left hand (*r*_s_ = -0.758; p = 0.011), but not with the right hand (*r*_s_ = -0.333; p = 0.347), nor with either hands on the RT task (-0.006 ≤ *r*_s_ ≤ 0.042; 0.907 ≤ p ≤ 0.987). In the CR group, bilateral Spearman analyses revealed non-significant correlations between condition 2 to condition 1 ratios of motor performance on the RT and GPB tasks with the right hand, and post- to pre-ratios of SICI in the left and right M1 (-0.382 ≤ *r*_s_ ≤ 0.406; 0.138 ≤ p ≤ 0.454).

{\displaystyle r_{s}}*rrr*

## Discussion

The present results show that Val66Val and Val66Met carriers do not differ on sensorimotor tasks involving speed, precision grip strength, and fine manual dexterity performed with the right or left hand. At the neurophysiological level, Val66Val and Val66Met carriers also did not differ on measures of corticospinal excitability and IHI from the left to right M1. Although SICI did not differ before and after motor practice in the Val66Val and Val66Met groups, Val66Val carriers exhibited a greater decrease in SICI in the bilateral M1 compared to Val66Met carriers following motor practice with both hands in an alternated sequence. SICI decrease in the Val66Val participants’ left M1 was correlated with motor improvement on the GPB task with the left hand. Interestingly, neither groups showed significant modulation of MEP amplitude or IHI when motor practice with the right hand is immediately preceded and followed by short periods of motor practice with the left hand on the same tasks. These results contrast with the increased MEP amplitudes, decreased SICI in left M1, and reduced IHI from the left to right M1 observed in the CR group, which performed the motor tasks with the right hand only. In addition, while TMS measures vary significantly between subjects, good to excellent test-retest reliability of RMT, MEP, and SICI were demonstrated with sample sizes similar to the present study (10; 10; 12; 15 subjects) [76–80]. This aspect is particularly important considering the main objective of the current experiment was to compare activity-dependent changes in TMS measures before and after motor practice between the Val66Val and the Val66Met groups. Our results show that the ICC on Pre- Post- RMT and MEP-rc were comparable to those reported in other experiments, as was SICI measured in the Val66Met group’s left M1 [76–80]. Other SICI measures were less reliable, particularly in the Val66Val group’s left M1 (poor) which is likely due to modulation through motor practice. Thus, the sample sizes were adequate for MEP-rc and SICI comparisons from time 1 (Pre) to time 2 (Post). However, lower reliabilities of the IHI measures may suggest a need for larger samples in future experiments.

The first objective of the present study was to characterize differences in learning and intermanual transfer of sensorimotor skills in Val66Val and Val66Met carriers. At the behavioral level, speed and fine motor dexterity in the left hand improved from condition 1 to condition 2 when no right-hand practice was present between conditions (CL group). These improvements did not differ from those observed in the Val66Val and Val66Met groups. Therefore, improvements in motor speed and fine manual dexterity with the left hand in Val66Val and Val66Met carriers, who performed motor tasks with their right hand for 30 minutes between conditions 1 and 2 with the left hand, are likely attributable to baseline practice with the left hand rather than intermanual transfer from the right to the left hand. Likewise, performance on the PGS task with the left hand did not improve from condition 1 to condition 2, irrespective of group, which again suggests the absence of intermanual transfer from the right dominant hand to the left non-dominant hand. These results contrast with previous studies that reported significant intermanual transfer of precision grip strength, motor speed, and complex fine manual dexterity [63–67, 82]. At the same time, reduced efficacy in intermanual transfer has also been shown with numerous sensorimotor protocols [83–87]. This variability suggests that intermanual transfer of sensorimotor information is a multidimensional mechanism sensitive to the nature of the task, availability of environmental feedback, and training schedule [84, 85, 88–91]. For example, intermanual transfer of sensorimotor information is more effective in tasks with increased reliance on higher-order perceptual and cognitive processes involved in motor planning and anticipation rather than simple dynamics such as force control [84, 85, 89]. Thus, the PGS task used in the present experiment, in the absence of object lifting, would not provide sufficient environmental feedback to foster sensorimotor memory formation, focusing instead on simple dynamic motor control. Indeed, intermanual transfer of simple dynamic motor skills, such as ballistic index finger abductions, relates to the level of effort during repetitive forceful contractions during a single session [48, 50, 55, 92, 93]. In comparison, rapid tapping tasks variants measure maximum contraction speed, which may be defined as inter-tap intervals, instead of maximal force contractions and acceleration [64, 65, 94]. Considering that intermanual transfer of motor speed was observed during intense training on rapid tapping tasks performed over multiple sessions [64, 65], it could be hypothesized that the intermanual transfer of non-forceful dynamic skills may be facilitated by consolidation and retention processes via repeated practice over several days [95]. A similar reasoning could be applied to the fine manual dexterity skills required for the GPB task, as previous experiments demonstrating significant intermanual transfer of complex fine dexterity skills were conducted over multiple sessions spread over several days [67, 96, 97].

Importantly, Val66Met participants did not differ at baseline or following motor practice from their Val66Val counterparts on motor tasks involving fast adaptations of low-force simple dynamic skills. Although replication in future experiments is required, the current results add to the growing body of evidence suggesting that the BDNF Val66Met polymorphism does not interfere with the execution and short-term learning of simple sensorimotor skills with the dominant hand [26–28, 98, 99] while showing similar findings for the left, non-dominant hand. Rather, the BDNF Val66Met polymorphism has been associated with impaired short-term learning and retention of motor skills on more complex tasks such as driving [27]. Hence, the impact of the Val66Met polymorphism on learning and retention of motor skills may be apparent when more complex visuomotor coordination is required, rather than unimanual movements restricted to simple dynamics and kinematics.

The second objective of the present study was to investigate the neurophysiological mechanisms that underlie sensorimotor learning and the intermanual transfer of sensorimotor information from the left to right M1 in Val66Val and Val66Met carriers. In the absence of significant intermanual transfer, motor improvements observed here likely result from practice with each hand separately, independently of practice with the other hand, although repeated practice over several sessions on similar tasks may lead to intermanual transfer [64, 65, 67, 96, 97]. The significant increase in left M1 corticospinal excitability following motor practice reported in Kleim et al. [26] was replicated in the present experiment in the CR group, which performed similar tasks with only their right hand. Consistent with previous studies, the CR group also exhibited reduced SICI in the left M1 and lower IHI from left to right M1 [45, 47, 55, 62]. However, neither Val66Val or Val66Met carriers displayed significant modulation of MEP amplitude in bilateral M1, or IHI from the left to right M1, after practicing with both hands in an alternated sequence. Furthermore, while alternated practice with both hands resulted in reduced SICI in the left and right and M1 of Val66Val carriers, practice with the right hand only did not elicit a decrease in SICI in the right M1. These results suggest that the decrease in SICI in the right M1 of Val66Val carriers is likely associated with the interaction effects of motor practice with both hands.

The discrepancy between neurophysiological activity elicited by practice with the right hand only, and with both hands in an alternated sequence, underlines complex neurophysiological interactions between the two M1s. Indeed, unilateral practice induces transient changes in cortical and corticospinal excitability in the M1 contralateral to the trained hand, as well as in the ipsilateral M1 [49, 53, 55, 56, 100]. These bidirectional interactions between the two M1s are mediated in part by interhemispheric inhibitory mechanisms via transcallosal pathways [52, 57–61]. It has been shown that unimanual practice is associated with decreased IHI from the M1 contralateral to the trained hand to the opposite M1 in procedural skill learning [47], pinch grip tasks [45], and submaximal force contractions over multiple sessions [62]. Moreover, the decrease in IHI correlates with cross education of muscle contractions and non-specific transfer on a procedural motor task [47, 62]. Long-term alterations of IHI have also been associated with extensive bimanual practice, as IHI is significantly reduced in trained musicians compared to non-musicians [101]. Taken together, these observations support the presence of reduced IHI from the trained M1 to the contralateral, untrained M1 following right-hand training found in the CR group. In addition, decreased IHI is associated with reduced SICI in the target M1 following motor training in protocols assessing intermanual transfer [45, 47]. Thus, it could be hypothesized that priming of the right M1 with the left-hand practice before and after training with the right hand facilitated SICI modulation in the right M1 in the Val66Val group. Similar to previous studies on intermanual transfer of motor skills, SICI decrease in the right M1 did not correlate with motor improvements with the contralateral left hand [47, 62], or with right-hand performance on the three tasks. Likewise, SICI decrease in the left M1 did not correlate with improvements in the contralateral right hand in the CR and Val66Val groups. However, our results showed a correlation between the magnitude of SICI decrease in Val66Val participants’ left M1 and increased motor improvement on the GPB task with the ipsilateral left hand. One possible interpretation is that SICI modulation in the left M1 occurring through practice with the right dominant hand is associated with intermanual transfer of low-force sensorimotor skill from the right dominant hand to the left dominant hand, thus underlying a potential role of the dominant M1 in motor skill transfer to the non-dominant M1. Observations of reduced SICI in the ipsilateral M1 during unilateral motor activity could support an association between both mechanisms in the context of intermanual transfer of motor skill [102, 103]. Future experiments are required to examine this relationship and the underlying mechanisms.

With regards to the absence of MEP amplitude modulation in Val66Val and Val66Met carriers, activation of the right M1 through left hand practice prior to, and after activation of the left M1 with right hand practice, may have interacted with corticospinal excitability levels in the left M1 and its ability to inhibit the opposite M1. For example, simultaneous practice with homologous muscles may decrease, or even suppress, MEP amplitudes in the M1 contralateral to the target hand compared to the unilateral practice condition alone [49], and others have failed to report significant changes in global MEP amplitudes following bimanual motor training [104]. The presence of an inhibitory interaction is further supported by reports of increased MEP amplitudes in the M1 contralateral to the trained hand on similar tasks in Val66Val carriers [26, 27]. As for the absence of IHI modulation in participants who performed the tasks with both hands, the available data clearly supports communication between M1s during unilateral motor tasks, as unilateral practice reduces IHI in both directions with adjusted CS intensities while either hand is active [59, 105, 106]. Likewise, rTMS to the left M1 reduces IHI in both directions, and IHI may be reduced or facilitated bidirectionally, or simultaneously enhanced in one direction and reduced in the other following transcranial direct current stimulation [106, 107]. Therefore, the execution of tasks with both hands in sequence could have triggered complex interactions in IHI resulting in the suppression of IHI from the left M1 to the right to M1.

Importantly, the present results show that the BDNF Val66Met polymorphism is associated with altered activity-dependent modulation of SICI in bilateral M1. It is also worth noting that the reported difference in SICI modulation between Val66Val and Val66Met carriers is not due to RMT or 1mV intensities because neither differed between groups, before or after motor training. These results are not surprising considering that BDNF regulates GABA-inhibitory synapse formation and maturation [16, 108–112]. For example, sensory deprivation leads to delayed GABA-ergic cell maturation in wild type mice, but not in BDNF over-expressing mice [108], and BDNF over-expression induces early maturation of GABA-ergic innervation and inhibition [109]. In the short-term, the BDNF and its precursor, pro-BDNF, modulate GABA-ergic synaptic plasticity, including presynaptic GABA release, and LTD presumably through NDMA-dependent transmission [9, 15, 16, 110, 111, 113, 114]. An underlying mechanism of fast changes in inhibitory transmission involves GABA-A receptor phosphorylation regulation associated with BDNF [5]. Moreover, sustained exposure to glutamatergic transmission and BDNF enhances long-lasting potentiation of GABA-A receptor related synaptic activity, which incidentally mediates SICI in the motor cortex [42, 110, 115, 116]. As such, the effects of the BDNF Val66Met polymorphism on cortical excitability have been extensively studied with TMS or tDCS plasticity-inducing protocols (see review by Chaied et al., 2014 [117]). Though the results have been inconsistent, the available data tend to confirm the association between altered cortical plasticity and the Val66Met polymorphism [117]. Specifically, GABA-A mediated SICI in the motor cortex was elevated at baseline, and following cathodal tDCS, in healthy Val66Met carriers compared to their Val66Val counterparts [118, 119]. Thus, the present results support the idea that BDNF Val66Met polymorphism is associated with altered short-term activity-dependent cortical excitability mechanisms in bilateral M1, despite the absence of differences in short-term motor learning of low force simple dynamic skills compared to Val66Val carriers. However, because SICI is associated with motor learning and intermanual transfer of procedural skill [39, 45–47], the absence of activity-dependant modulation of SICI provides additional support for the short-term learning and retention problems on more complex tasks [27, 68], as well as impaired intermanual transfer of procedural skill reported in Val66Met carriers [29].

### Limits

Non-normal distributions are probably due to artefacts related to the small sample sizes used in the current study, which was determined *a priori* based on previous studies on the associations between the Val66Met genotype, cortical plasticity, and motor learning (7, 8, 11, and 12 Val66Met subjects) [26–28, 120]. Notably, the analyses comprised of non-normal transformed variables did not conclude to significant main effects or interactions. While simulation studies with ANOVA tests have shown that the false positive rate is not much affected by the violation of the assumption of normal distribution [121], there is also a possibility of an overestimation of type 2 errors under such conditions [122]. Therefore, the negative results in this study have been interpreted with care, not by suggesting the absence of effects in the general population but rather by highlighting similarities with the existing literature. Importantly, the standardized residuals distribution of the variables involved in the SICI analysis comparing the Val66Val and the Val66Met participants did not differ from normal distribution. Therefore, the validity of this study’s main finding, namely, the differential activity-dependent modulation of bi-hemispheric SICI between the Val66Val and the Val66Met participants, is supported. Nevertheless, replication in future experiments is required.

## Supporting information

S1 Minimal Data Set (XLSX)

## References

[1] Carvalho A.L., et al, Role of the brain-derived neurotrophic factor at glutamatergic synapses. Br J Pharmacol, 2008 153 Suppl 1: p. S310–24. 18059328 10.1038/sj.bjp.0707509 PMC2268077

[2] Zhou X.F., et al, Distribution and localization of pro-brain-derived neurotrophic factor-like immunoreactivity in the peripheral and central nervous system of the adult rat. J Neurochem, 2004 91(3): p. 704–15. doi: 10.1111/j.1471-4159.2004.02775.x 15485500 10.1111/j.1471-4159.2004.02775.x

[3] Baldelli P., et al, Brain-derived neurotrophic factor enhances GABA release probability and nonuniform distribution of N- and P/Q-type channels on release sites of hippocampal inhibitory synapses. J Neurosci, 2005 25(13): p. 3358–68. doi: 10.1523/JNEUROSCI.4227-04.2005 15800191 10.1523/JNEUROSCI.4227-04.2005 PMC6724891

[4] Frerking M., Malenka R.C., and Nicoll R.A., Brain-derived neurotrophic factor (BDNF) modulates inhibitory, but not excitatory, transmission in the CA1 region of the hippocampus. J Neurophysiol, 1998 80(6): p. 3383–6. doi: 10.1152/jn.1998.80.6.3383 9862938 10.1152/jn.1998.80.6.3383

[5] Jovanovic J.N., et al, Brain-derived neurotrophic factor modulates fast synaptic inhibition by regulating GABA(A) receptor phosphorylation, activity, and cell-surface stability. J Neurosci, 2004 24(2): p. 522–30. doi: 10.1523/JNEUROSCI.3606-03.2004 14724252 10.1523/JNEUROSCI.3606-03.2004 PMC6729993

[6] Lessmann V., Gottmann K., and Malcangio M., Neurotrophin secretion: current facts and future prospects. Prog Neurobiol, 2003 69(5): p. 341–74. 12787574 10.1016/s0301-0082(03)00019-4

[7] Matsumoto T., et al, Brain-derived neurotrophic factor-induced potentiation of glutamate and GABA release: different dependency on signaling pathways and neuronal activity. Mol Cell Neurosci, 2006 31(1): p. 70–84. doi: 10.1016/j.mcn.2005.09.002 16214365 10.1016/j.mcn.2005.09.002

[8] Poo M.M., Neurotrophins as synaptic modulators. Nat Rev Neurosci, 2001 2(1): p. 24–32. doi: 10.1038/35049004 11253356 10.1038/35049004

[9] Wardle R.A. and Poo M.M., Brain-derived neurotrophic factor modulation of GABAergic synapses by postsynaptic regulation of chloride transport. J Neurosci, 2003 23(25): p. 8722–32. 14507972 10.1523/JNEUROSCI.23-25-08722.2003 PMC6740427

[10] Aicardi G., et al, Induction of long-term potentiation and depression is reflected by corresponding changes in secretion of endogenous brain-derived neurotrophic factor. Proc Natl Acad Sci U S A, 2004 101(44): p. 15788–92. doi: 10.1073/pnas.0406960101 15505222 10.1073/pnas.0406960101 PMC524856

[11] Figurov A., et al, Regulation of synaptic responses to high-frequency stimulation and LTP by neurotrophins in the hippocampus. Nature, 1996 381(6584): p. 706–9. doi: 10.1038/381706a0 8649517 10.1038/381706a0

[12] Lu B., BDNF and activity-dependent synaptic modulation. Learn Mem, 2003 10(2): p. 86–98. doi: 10.1101/lm.54603 12663747 10.1101/lm.54603 PMC5479144

[13] McAllister A.K., Katz L.C., and Lo D.C., Neurotrophins and synaptic plasticity. Annu Rev Neurosci, 1999 22: p. 295–318. doi: 10.1146/annurev.neuro.22.1.295 10202541 10.1146/annurev.neuro.22.1.295

[14] Thoenen H., Neurotrophins and activity-dependent plasticity. Prog Brain Res, 2000 128: p. 183–91. doi: 10.1016/S0079-6123(00)28016-3 11105678 10.1016/S0079-6123(00)28016-3

[15] Woo N.H., et al, Activation of p75NTR by proBDNF facilitates hippocampal long-term depression. Nat Neurosci, 2005 8(8): p. 1069–77. doi: 10.1038/nn1510 16025106 10.1038/nn1510

[16] Park H. and Poo M.M., Neurotrophin regulation of neural circuit development and function. Nat Rev Neurosci, 2013 14(1): p. 7–23. doi: 10.1038/nrn3379 23254191 10.1038/nrn3379

[17] Hess G. and Donoghue J.P., Long-term potentiation of horizontal connections provides a mechanism to reorganize cortical motor maps. J Neurophysiol, 1994 71(6): p. 2543–7. doi: 10.1152/jn.1994.71.6.2543 7931533 10.1152/jn.1994.71.6.2543

[18] Rioult-Pedotti M.S., et al, Strengthening of horizontal cortical connections following skill learning. Nat Neurosci, 1998 1(3): p. 230–4. doi: 10.1038/678 10195148 10.1038/678

[19] Sanes J.N. and Donoghue J.P., Plasticity and primary motor cortex. Annu Rev Neurosci, 2000 23: p. 393–415. doi: 10.1146/annurev.neuro.23.1.393 10845069 10.1146/annurev.neuro.23.1.393

[20] Karabanov A., et al, Consensus Paper: Probing Homeostatic Plasticity of Human Cortex With Non-invasive Transcranial Brain Stimulation. Brain Stimul, 2015 8(5): p. 993–1006. 26598772 10.1016/j.brs.2015.06.017

[21] Shimizu E., Hashimoto K., and Iyo M., Ethnic difference of the BDNF 196G/A (val66met) polymorphism frequencies: the possibility to explain ethnic mental traits. Am J Med Genet B Neuropsychiatr Genet, 2004 126B(1): p. 122–3. doi: 10.1002/ajmg.b.20118 15048661 10.1002/ajmg.b.20118

[22] Carballedo A., et al, Reduced fractional anisotropy in the uncinate fasciculus in patients with major depression carrying the met-allele of the Val66Met brain-derived neurotrophic factor genotype. Am J Med Genet B Neuropsychiatr Genet, 2012 159B(5): p. 537–48. doi: 10.1002/ajmg.b.32060 22585743 10.1002/ajmg.b.32060

[23] Kennedy K.M., et al, BDNF Val66Met polymorphism influences age differences in microstructure of the Corpus Callosum. Front Hum Neurosci, 2009 3: p. 19 doi: 10.3389/neuro.09.019.2009 19738930 10.3389/neuro.09.019.2009 PMC2737488

[24] Ninan I., et al, The BDNF Val66Met polymorphism impairs NMDA receptor-dependent synaptic plasticity in the hippocampus. J Neurosci, 2010 30(26): p. 8866–70. doi: 10.1523/JNEUROSCI.1405-10.2010 20592208 10.1523/JNEUROSCI.1405-10.2010 PMC2911131

[25] Pattwell S.S., et al, The BDNF Val66Met polymorphism impairs synaptic transmission and plasticity in the infralimbic medial prefrontal cortex. J Neurosci, 2012 32(7): p. 2410–21. doi: 10.1523/JNEUROSCI.5205-11.2012 22396415 10.1523/JNEUROSCI.5205-11.2012 PMC3532006

[26] Kleim J.A., et al, BDNF val66met polymorphism is associated with modified experience-dependent plasticity in human motor cortex. Nat Neurosci, 2006 9(6): p. 735–7. doi: 10.1038/nn1699 16680163 10.1038/nn1699

[27] McHughen S.A., et al, BDNF val66met polymorphism influences motor system function in the human brain. Cereb Cortex, 2010 20(5): p. 1254–62. doi: 10.1093/cercor/bhp189 19745020 10.1093/cercor/bhp189 PMC2852510

[28] McHughen S.A., et al, Intense training overcomes effects of the Val66Met BDNF polymorphism on short-term plasticity. Exp Brain Res, 2011 213(4): p. 415–22. doi: 10.1007/s00221-011-2791-z 21769545 10.1007/s00221-011-2791-z

[29] Morin-Moncet O., et al, BDNF Val66Met polymorphism is associated with abnormal interhemispheric transfer of a newly acquired motor skill. J Neurophysiol, 2014 111(10): p. 2094–102. doi: 10.1152/jn.00388.2013 24572097 10.1152/jn.00388.2013

[30] Pascual-Leone A., et al, Modulation of muscle responses evoked by transcranial magnetic stimulation during the acquisition of new fine motor skills. J Neurophysiol, 1995 74(3): p. 1037–45. doi: 10.1152/jn.1995.74.3.1037 7500130 10.1152/jn.1995.74.3.1037

[31] Perez M.A., *The Functional Role of Interhemispheric Interactions in Human Motor Control*, in *Cortical Connectivity: Brain Stimulation for Assessing and Modulating Cortical Connectivity and Function*, Chen R. and Rothwell J.C., Editors. 2012, Springer Berlin Heidelberg: Berlin, Heidelberg p. 165–181.

[32] Reis J., et al, Contribution of transcranial magnetic stimulation to the understanding of cortical mechanisms involved in motor control. J Physiol, 2008 586(2): p. 325–51. doi: 10.1113/jphysiol.2007.144824 17974592 10.1113/jphysiol.2007.144824 PMC2375593

[33] Ruddy K.L. and Carson R.G., Neural pathways mediating cross education of motor function. Front Hum Neurosci, 2013 7: p. 397 doi: 10.3389/fnhum.2013.00397 23908616 10.3389/fnhum.2013.00397 PMC3725409

[34] Ziemann U., Ilic T.V., and Jung P., Long-term potentiation (LTP)-like plasticity and learning in human motor cortex—investigations with transcranial magnetic stimulation (TMS). Suppl Clin Neurophysiol, 2006 59: p. 19–25. 16893088 10.1016/s1567-424x(09)70007-8

[35] Morin-Moncet O., et al, Action Video Game Playing Is Reflected In Enhanced Visuomotor Performance and Increased Corticospinal Excitability. PLoS One, 2016 11(12): p. e0169013 doi: 10.1371/journal.pone.0169013 28005989 10.1371/journal.pone.0169013 PMC5179116

[36] Hallett M., Transcranial Magnetic Stimulation: A Primer. Neuron, 2007 55(2): p. 187–199. doi: 10.1016/j.neuron.2007.06.026 17640522 10.1016/j.neuron.2007.06.026

[37] Karabanov A. and Siebner H.R., Unravelling homeostatic interactions in inhibitory and excitatory networks in human motor cortex. J Physiol, 2012 590(22): p. 5557–8. doi: 10.1113/jphysiol.2012.244749 23154853 10.1113/jphysiol.2012.244749 PMC3528974

[38] Murakami T., et al, Homeostatic metaplasticity of corticospinal excitatory and intracortical inhibitory neural circuits in human motor cortex. J Physiol, 2012 590(22): p. 5765–81. doi: 10.1113/jphysiol.2012.238519 22930265 10.1113/jphysiol.2012.238519 PMC3528990

[39] Coxon J.P., Peat N.M., and Byblow W.D., Primary motor cortex disinhibition during motor skill learning. J Neurophysiol, 2014 112(1): p. 156–64. doi: 10.1152/jn.00893.2013 24717346 10.1152/jn.00893.2013

[40] Ilic T.V., et al, Short-interval paired-pulse inhibition and facilitation of human motor cortex: the dimension of stimulus intensity. J Physiol, 2002 545(Pt 1): p. 153–67. doi: 10.1113/jphysiol.2002.030122 12433957 10.1113/jphysiol.2002.030122 PMC2290644

[41] Kujirai T., et al, Corticocortical inhibition in human motor cortex. J Physiol, 1993 471: p. 501–19. 8120818 10.1113/jphysiol.1993.sp019912 PMC1143973

[42] Stagg C.J., et al, Relationship between physiological measures of excitability and levels of glutamate and GABA in the human motor cortex. J Physiol, 2011 589(Pt 23): p. 5845–55. doi: 10.1113/jphysiol.2011.216978 22005678 10.1113/jphysiol.2011.216978 PMC3249054

[43] Ziemann U., Rothwell J.C., and Ridding M.C., Interaction between intracortical inhibition and facilitation in human motor cortex. J Physiol, 1996 496 (Pt 3): p. 873–81. 8930851 10.1113/jphysiol.1996.sp021734 PMC1160871

[44] Sugawara K., et al, Functional plasticity of surround inhibition in the motor cortex during single finger contraction training. Neuroreport, 2012 23(11): p. 663–7. doi: 10.1097/WNR.0b013e3283556522 22643236 10.1097/WNR.0b013e3283556522

[45] Camus M., et al, Mechanisms controlling motor output to a transfer hand after learning a sequential pinch force skill with the opposite hand. Clin Neurophysiol, 2009 120(10): p. 1859–65. doi: 10.1016/j.clinph.2009.08.013 19766535 10.1016/j.clinph.2009.08.013 PMC2767461

[46] Cirillo J., Todd G., and Semmler J.G., Corticomotor excitability and plasticity following complex visuomotor training in young and old adults. Eur J Neurosci, 2011 34(11): p. 1847–56. doi: 10.1111/j.1460-9568.2011.07870.x 22004476 10.1111/j.1460-9568.2011.07870.x

[47] Perez M.A., et al, Neurophysiological mechanisms involved in transfer of procedural knowledge. J Neurosci, 2007 27(5): p. 1045–53. doi: 10.1523/JNEUROSCI.4128-06.2007 17267558 10.1523/JNEUROSCI.4128-06.2007 PMC6673204

[48] Carroll T.J., et al, Unilateral practice of a ballistic movement causes bilateral increases in performance and corticospinal excitability. J Appl Physiol (1985), 2008. 104(6): p. 1656–64. 10.1152/japplphysiol.01351.2007 18403447

[49] Ghacibeh G.A., et al, Ipsilateral motor activation during unimanual and bimanual motor tasks. Clin Neurophysiol, 2007 118(2): p. 325–32. doi: 10.1016/j.clinph.2006.10.003 17095289 10.1016/j.clinph.2006.10.003

[50] Hess C.W., Mills K.R., and Murray N.M., Magnetic stimulation of the human brain: facilitation of motor responses by voluntary contraction of ipsilateral and contralateral muscles with additional observations on an amputee. Neurosci Lett, 1986 71(2): p. 235–40. 3785745 10.1016/0304-3940(86)90565-3

[51] Hortobagyi T., et al, Changes in segmental and motor cortical output with contralateral muscle contractions and altered sensory inputs in humans. J Neurophysiol, 2003 90(4): p. 2451–9. doi: 10.1152/jn.01001.2002 14534271 10.1152/jn.01001.2002

[52] Kobayashi M., et al, Ipsilateral motor cortex activation on functional magnetic resonance imaging during unilateral hand movements is related to interhemispheric interactions. Neuroimage, 2003 20(4): p. 2259–70. 14683727 10.1016/s1053-8119(03)00220-9

[53] Liang N., et al, Further evidence for excitability changes in human primary motor cortex during ipsilateral voluntary contractions. Neurosci Lett, 2008 433(2): p. 135–40. doi: 10.1016/j.neulet.2007.12.058 18261851 10.1016/j.neulet.2007.12.058

[54] Muellbacher W., et al, Changes in motor cortex excitability during ipsilateral hand muscle activation in humans. Clin Neurophysiol, 2000 111(2): p. 344–9. 10680571 10.1016/s1388-2457(99)00243-6

[55] Perez M.A. and Cohen L.G., Mechanisms underlying functional changes in the primary motor cortex ipsilateral to an active hand. J Neurosci, 2008 28(22): p. 5631–40. doi: 10.1523/JNEUROSCI.0093-08.2008 18509024 10.1523/JNEUROSCI.0093-08.2008 PMC2440822

[56] Ziemann U. and Hallett M., Hemispheric asymmetry of ipsilateral motor cortex activation during unimanual motor tasks: further evidence for motor dominance. Clin Neurophysiol, 2001 112(1): p. 107–13. 11137667 10.1016/s1388-2457(00)00502-2

[57] Ferbert A., et al, Interhemispheric inhibition of the human motor cortex. J Physiol, 1992 453: p. 525–46. 1464843 10.1113/jphysiol.1992.sp019243 PMC1175572

[58] Kobayashi M., et al, Repetitive TMS of the motor cortex improves ipsilateral sequential simple finger movements. Neurology, 2004 62(1): p. 91–8. 14718704 10.1212/wnl.62.1.91

[59] Nelson A.J., et al, Bi-directional interhemispheric inhibition during unimanual sustained contractions. BMC Neurosci, 2009 10: p. 31 doi: 10.1186/1471-2202-10-31 19344522 10.1186/1471-2202-10-31 PMC2669479

[60] Ni Z., et al, Two phases of interhemispheric inhibition between motor related cortical areas and the primary motor cortex in human. Cereb Cortex, 2009 19(7): p. 1654–65. doi: 10.1093/cercor/bhn201 19015374 10.1093/cercor/bhn201

[61] Ragert P., et al, Modulation of effects of intermittent theta burst stimulation applied over primary motor cortex (M1) by conditioning stimulation of the opposite M1. J Neurophysiol, 2009 102(2): p. 766–73. doi: 10.1152/jn.00274.2009 19474173 10.1152/jn.00274.2009 PMC2724345

[62] Hortobagyi T., et al, Interhemispheric plasticity in humans. Med Sci Sports Exerc, 2011 43(7): p. 1188–99. doi: 10.1249/MSS.0b013e31820a94b8 21200340 10.1249/MSS.0b013e31820a94b8 PMC4137570

[63] Chase C. and Seidler R., Degree of handedness affects intermanual transfer of skill learning. Exp Brain Res, 2008 190(3): p. 317–28. doi: 10.1007/s00221-008-1472-z 18592225 10.1007/s00221-008-1472-z PMC2570758

[64] Koeneke S., et al, Extensive training of elementary finger tapping movements changes the pattern of motor cortex excitability. Exp Brain Res, 2006 174(2): p. 199–209. doi: 10.1007/s00221-006-0440-8 16604315 10.1007/s00221-006-0440-8

[65] Koeneke S., et al, Transfer effects of practice for simple alternating movements. J Mot Behav, 2009 41(4): p. 347–55. doi: 10.3200/JMBR.41.4.347-356 19508961 10.3200/JMBR.41.4.347-356

[66] Liang N., et al, Effects of intermanual transfer induced by repetitive precision grip on input-output properties of untrained contralateral limb muscles. Exp Brain Res, 2007 182(4): p. 459–67. doi: 10.1007/s00221-007-1004-2 17562034 10.1007/s00221-007-1004-2

[67] Schulze K., Luders E., and Jancke L., Intermanual transfer in a simple motor task. Cortex, 2002 38(5): p. 805–15. 12507049 10.1016/s0010-9452(08)70047-9

[68] Smolders R., et al, BDNF Val66Met polymorphism interacts with sex to influence bimanual motor control in healthy humans. Brain Behav, 2012 2(6): p. 726–31. doi: 10.1002/brb3.83 23170235 10.1002/brb3.83 PMC3500459

[69] Rossi S., et al, Safety, ethical considerations, and application guidelines for the use of transcranial magnetic stimulation in clinical practice and research. Clin Neurophysiol, 2009 120(12): p. 2008–39. doi: 10.1016/j.clinph.2009.08.016 19833552 10.1016/j.clinph.2009.08.016 PMC3260536

[70] Petersen R.C., et al, Vitamin E and donepezil for the treatment of mild cognitive impairment. N Engl J Med, 2005 352(23): p. 2379–88. doi: 10.1056/NEJMoa050151 15829527 10.1056/NEJMoa050151

[71] Rossini P.M., et al, Non-invasive electrical and magnetic stimulation of the brain, spinal cord and roots: basic principles and procedures for routine clinical application. Report of an IFCN committee. Electroencephalogr Clin Neurophysiol, 1994 91(2): p. 79–92. 7519144 10.1016/0013-4694(94)90029-9

[72] Peurala S.H., et al, Interference of short-interval intracortical inhibition (SICI) and short-interval intracortical facilitation (SICF). Clin Neurophysiol, 2008 119(10): p. 2291–7. doi: 10.1016/j.clinph.2008.05.031 18723394 10.1016/j.clinph.2008.05.031

[73] Tremblay S., et al, Relationship between transcranial magnetic stimulation measures of intracortical inhibition and spectroscopy measures of GABA and glutamate+glutamine. Journal of Neurophysiology, 2013 109(5): p. 1343–1349. doi: 10.1152/jn.00704.2012 23221412 10.1152/jn.00704.2012 PMC3602833

[74] Fisher R.J., et al, Two phases of intracortical inhibition revealed by transcranial magnetic threshold tracking. Experimental Brain Research, 2002 143(2): p. 240–248. doi: 10.1007/s00221-001-0988-2 11880900 10.1007/s00221-001-0988-2

[75] Roshan L., Paradiso G.O., and Chen R., Two phases of short-interval intracortical inhibition. Experimental Brain Research, 2003 151(3): p. 330–337. doi: 10.1007/s00221-003-1502-9 12802553 10.1007/s00221-003-1502-9

[76] Biabani M., et al, The minimal number of TMS trials required for the reliable assessment of corticospinal excitability, short interval intracortical inhibition, and intracortical facilitation. Neurosci Lett, 2018 674: p. 94–100. doi: 10.1016/j.neulet.2018.03.026 29551425 10.1016/j.neulet.2018.03.026

[77] Fleming M.K., et al, The effect of coil type and navigation on the reliability of transcranial magnetic stimulation. IEEE Trans Neural Syst Rehabil Eng, 2012 20(5): p. 617–25. doi: 10.1109/TNSRE.2012.2202692 22695363 10.1109/TNSRE.2012.2202692

[78] Maeda F., et al, Inter- and intra-individual variability of paired-pulse curves with transcranial magnetic stimulation (TMS). Clin Neurophysiol, 2002 113(3): p. 376–82. 11897538 10.1016/s1388-2457(02)00008-1

[79] Mooney R.A., Cirillo J., and Byblow W.D., GABA and primary motor cortex inhibition in young and older adults: a multimodal reliability study. J Neurophysiol, 2017 118(1): p. 425–433. doi: 10.1152/jn.00199.2017 28424294 10.1152/jn.00199.2017 PMC5506262

[80] Ngomo S., et al, Comparison of transcranial magnetic stimulation measures obtained at rest and under active conditions and their reliability. J Neurosci Methods, 2012 205(1): p. 65–71. doi: 10.1016/j.jneumeth.2011.12.012 22227444 10.1016/j.jneumeth.2011.12.012

[81] Cicchetti D.V. and Sparrow S.A., Developing criteria for establishing interrater reliability of specific items: applications to assessment of adaptive behavior. Am J Ment Defic, 1981 86(2): p. 127–37. 7315877

[82] Gordon A.M., Forssberg H., and Iwasaki N., Formation and lateralization of internal representations underlying motor commands during precision grip. Neuropsychologia, 1994 32(5): p. 555–68. 8084414 10.1016/0028-3932(94)90144-9

[83] Karni A., et al, Functional MRI evidence for adult motor cortex plasticity during motor skill learning. Nature, 1995 377(6545): p. 155–8. doi: 10.1038/377155a0 7675082 10.1038/377155a0

[84] Teixeira L.A., Timing and force components in bilateral transfer of learning. Brain Cogn, 2000 44(3): p. 455–69. doi: 10.1006/brcg.1999.1205 11104537 10.1006/brcg.1999.1205

[85] Teixeira L.A. and Caminha L.Q., Intermanual transfer of force control is modulated by asymmetry of muscular strength. Exp Brain Res, 2003 149(3): p. 312–9. doi: 10.1007/s00221-002-1363-7 12632233 10.1007/s00221-002-1363-7

[86] Park J.H. and Shea C.H., Effector independence. J Mot Behav, 2002 34(3): p. 253–70. doi: 10.1080/00222890209601944 19260176 10.1080/00222890209601944

[87] Salimi I., et al, Specificity of internal representations underlying grasping. J Neurophysiol, 2000 84(5): p. 2390–7. doi: 10.1152/jn.2000.84.5.2390 11067981 10.1152/jn.2000.84.5.2390

[88] Hollerbach J.M., *Fundamentals of motor behavior*, in *Visual cognition and action (vol*.*2)*, Daniel N.O., Stephen Michael K., and John M.H., Editors. 1990, MIT Press p. 151–182.

[89] Imamizu H. and Shimojo S., The locus of visual-motor learning at the task or manipulator level: implications from intermanual transfer. J Exp Psychol Hum Percept Perform, 1995 21(4): p. 719–33. 7643045 10.1037//0096-1523.21.4.719

[90] Ioffe M., et al, Coordination between posture and movement in a bimanual load-lifting task: is there a transfer? Exp Brain Res, 1996 109(3): p. 450–6. 8817275 10.1007/BF00229629

[91] Sathian K. and Zangaladze A., Perceptual learning in tactile hyperacuity: complete intermanual transfer but limited retention. Exp Brain Res, 1998 118(1): p. 131–4. 9547071 10.1007/s002210050263

[92] Lee M., et al, The ipsilateral motor cortex contributes to cross-limb transfer of performance gains after ballistic motor practice. J Physiol, 2010 588(Pt 1): p. 201–12. doi: 10.1113/jphysiol.2009.183855 19917563 10.1113/jphysiol.2009.183855 PMC2821559

[93] Parikh P.J. and Cole K.J., Transfer of learning between hands to handle a novel object in old age. Exp Brain Res, 2013 227(1): p. 9–18. doi: 10.1007/s00221-013-3451-2 23595702 10.1007/s00221-013-3451-2

[94] Fujii S., et al, Wrist muscle activity during rapid unimanual tapping with a drumstick in drummers and nondrummers. Motor Control, 2009 13(3): p. 237–50. 19799164 10.1123/mcj.13.3.237

[95] Doyon J., Penhune V., and Ungerleider L.G., Distinct contribution of the cortico-striatal and cortico-cerebellar systems to motor skill learning. Neuropsychologia, 2003 41(3): p. 252–62. 12457751 10.1016/s0028-3932(02)00158-6

[96] Ausenda C. and Carnovali M., Transfer of motor skill learning from the healthy hand to the paretic hand in stroke patients: a randomized controlled trial. Eur J Phys Rehabil Med, 2011 47(3): p. 417–25. 21555982

[97] Pereira E.A., Raja K., and Gangavalli R., Effect of training on interlimb transfer of dexterity skills in healthy adults. Am J Phys Med Rehabil, 2011 90(1): p. 25–34. doi: 10.1097/PHM.0b013e3181fc7f6f 20975521 10.1097/PHM.0b013e3181fc7f6f

[98] Cirillo J., et al, Differential modulation of motor cortex excitability in BDNF Met allele carriers following experimentally induced and use-dependent plasticity. Eur J Neurosci, 2012 36(5): p. 2640–9. doi: 10.1111/j.1460-9568.2012.08177.x 22694150 10.1111/j.1460-9568.2012.08177.x

[99] Li Voti P., et al, Correlation between cortical plasticity, motor learning and BDNF genotype in healthy subjects. Exp Brain Res, 2011 212(1): p. 91–9. doi: 10.1007/s00221-011-2700-5 21537966 10.1007/s00221-011-2700-5

[100] Uematsu A., et al, Asymmetrical modulation of corticospinal excitability in the contracting and resting contralateral wrist flexors during unilateral shortening, lengthening and isometric contractions. Exp Brain Res, 2010 206(1): p. 59–69. doi: 10.1007/s00221-010-2397-x 20730420 10.1007/s00221-010-2397-x

[101] Ridding M.C., Brouwer B., and Nordstrom M.A., Reduced interhemispheric inhibition in musicians. Exp Brain Res, 2000 133(2): p. 249–53. 10968226 10.1007/s002210000428

[102] Howatson G., et al, Ipsilateral motor cortical responses to TMS during lengthening and shortening of the contralateral wrist flexors. The European journal of neuroscience, 2011 33(5): p. 978–990. doi: 10.1111/j.1460-9568.2010.07567.x 21219480 10.1111/j.1460-9568.2010.07567.x PMC3075420

[103] Morishita T., et al, Increased excitability and reduced intracortical inhibition in the ipsilateral primary motor cortex during a fine-motor manipulation task. Brain Research, 2011 1371: p. 65–73. doi: 10.1016/j.brainres.2010.11.049 21093420 10.1016/j.brainres.2010.11.049

[104] Neva J.L., et al, Selective modulation of left primary motor cortex excitability after continuous theta burst stimulation to right primary motor cortex and bimanual training. Behav Brain Res, 2014 269: p. 138–46. doi: 10.1016/j.bbr.2014.04.041 24786332 10.1016/j.bbr.2014.04.041

[105] Sattler V., et al, Interhemispheric inhibition in human wrist muscles. Exp Brain Res, 2012 221(4): p. 449–58. doi: 10.1007/s00221-012-3187-4 22923264 10.1007/s00221-012-3187-4

[106] Pal P.K., et al, Effect of low-frequency repetitive transcranial magnetic stimulation on interhemispheric inhibition. J Neurophysiol, 2005 94(3): p. 1668–75. doi: 10.1152/jn.01306.2004 15872061 10.1152/jn.01306.2004

[107] Tazoe T., et al, Polarity specific effects of transcranial direct current stimulation on interhemispheric inhibition. PLoS One, 2014 9(12): p. e114244 doi: 10.1371/journal.pone.0114244 25478912 10.1371/journal.pone.0114244 PMC4257682

[108] Gianfranceschi L., et al, Visual cortex is rescued from the effects of dark rearing by overexpression of BDNF. Proc Natl Acad Sci U S A, 2003 100(21): p. 12486–91. doi: 10.1073/pnas.1934836100 14514885 10.1073/pnas.1934836100 PMC218784

[109] Huang Z.J., et al, BDNF regulates the maturation of inhibition and the critical period of plasticity in mouse visual cortex. Cell, 1999 98(6): p. 739–55. 10499792 10.1016/s0092-8674(00)81509-3

[110] Kuczewski N., et al, Spontaneous glutamatergic activity induces a BDNF-dependent potentiation of GABAergic synapses in the newborn rat hippocampus. J Physiol, 2008 586(21): p. 5119–28. doi: 10.1113/jphysiol.2008.158550 18772203 10.1113/jphysiol.2008.158550 PMC2652155

[111] Langlois A., et al, NMDA-dependent switch of proBDNF actions on developing GABAergic synapses. Cereb Cortex, 2013 23(5): p. 1085–96. doi: 10.1093/cercor/bhs071 22510533 10.1093/cercor/bhs071 PMC3615346

[112] Yamada M.K., et al, Brain-derived neurotrophic factor promotes the maturation of GABAergic mechanisms in cultured hippocampal neurons. J Neurosci, 2002 22(17): p. 7580–5. 12196581 10.1523/JNEUROSCI.22-17-07580.2002 PMC6757965

[113] Cheng Q., Song S.H., and Augustine G.J., Calcium-Dependent and Synapsin-Dependent Pathways for the Presynaptic Actions of BDNF. Front Cell Neurosci, 2017 11: p. 75 doi: 10.3389/fncel.2017.00075 28392759 10.3389/fncel.2017.00075 PMC5364187

[114] Woo N. and Lu B., *BDNF in synaptic plasticity and memory* Intracellular Communication In The Nervous System. Bethesda, Maryland, USA: NIH, 2009: p. 135–143.

[115] Ziemann U., TMS and drugs. Clin Neurophysiol, 2004 115(8): p. 1717–29. doi: 10.1016/j.clinph.2004.03.006 15261850 10.1016/j.clinph.2004.03.006

[116] Ziemann U., et al, TMS and drugs revisited 2014. Clin Neurophysiol, 2015 126(10): p. 1847–68. doi: 10.1016/j.clinph.2014.08.028 25534482 10.1016/j.clinph.2014.08.028

[117] Chaieb L., et al, Brain-derived neurotrophic factor: its impact upon neuroplasticity and neuroplasticity inducing transcranial brain stimulation protocols. Neurogenetics, 2014 15(1): p. 1–11. doi: 10.1007/s10048-014-0393-1 24567226 10.1007/s10048-014-0393-1

[118] Cash R., et al, Influence of the BDNF Val66Met polymorphism on the balance of excitatory and inhibitory neurotransmission and relationship to plasticity in human cortex. Brain Stimulation: Basic, Translational, and Clinical Research in Neuromodulation, 2017 10(2): p. 502.

[119] Strube W., et al, BDNF-Val66Met-polymorphism impact on cortical plasticity in schizophrenia patients: a proof-of-concept study. Int J Neuropsychopharmacol, 2014 18(4). 10.1093/ijnp/pyu040 PMC4360229 25612896

[120] Cheeran B., et al, A common polymorphism in the brain-derived neurotrophic factor gene (BDNF) modulates human cortical plasticity and the response to rTMS. J Physiol, 2008 586(23): p. 5717–25. doi: 10.1113/jphysiol.2008.159905 18845611 10.1113/jphysiol.2008.159905 PMC2655403

[121] McDonald J.H., *Handbook of Biological Statistics* 3 ed 2014, Baltimore, Maryland: Sparky House Publishing.

[122] Fayers P., Alphas, betas and skewy distributions: two ways of getting the wrong answer. Advances in Health Sciences Education, 2011 16(3): p. 291–296. doi: 10.1007/s10459-011-9283-6 21400008 10.1007/s10459-011-9283-6 PMC3139856

